# Susceptibility of spotted doves (*Streptopelia chinensis*) to experimental infection with the severe fever with thrombocytopenia syndrome phlebovirus

**DOI:** 10.1371/journal.pntd.0006982

**Published:** 2019-07-05

**Authors:** Zhifeng Li, Changjun Bao, Jianli Hu, Chengfeng Gao, Nan Zhang, Huo Xiang, Carol J. Cardona, Zheng Xing

**Affiliations:** 1 Nanjing University Medical School and Jiangsu Provincial Key Laboratory of Medicine, Nanjing, China; 2 Jiangsu Provincial Center for Disease Prevention and Control, Nanjing, China; 3 College of Veterinary Medicine, University of Minnesota at Twin Cities, Saint Paul, Minnesota, United States of America; Fort Collins, UNITED STATES

## Abstract

**Background:**

Severe fever with thrombocytopenia syndrome virus (SFTSV), an emerging human pathogen naturally transmitted by ticks, has spread widely since it was first detected in 2010. Although SFTSV-specific antibodies have been detected in wild birds, these natural reservoir and amplifying hosts for the virus have not been well studied.

**Methodology/Principle findings:**

Here we report an experimental infection of spotted doves (*Streptopelia chinensis*) with two strains of SFTSV, JS2010-14, a Chinese lineage strain, and JS2014-16, a Japanese lineage strain, which represent the main viral genotypes currently circulating in East Asia. In these studies, we have determined that spotted doves are susceptible to SFTSV and the severity of the viremia is dose-dependent. When challenged with 10^7^ and 10^5^ PFU, all doves developed viremia which peaked 3–5 days post infection (dpi). Only a subset (25–62.5%) of the birds developed viremia when challenged at 10^3^ PFU. Virulence of SFTSV in spotted doves was strain dependent. Infection with 10^7^ PFU of strain JS2014-16 resulted in 12.5% mortality over 6.8 days and mean peak viremia titers of 10^6.9^ PFU/mL in experimentally inoculated birds. All doves inoculated with 10^7^ PFU of the JS2010-14 strain survived infection with relatively lower mean viremia titers (10^5.6^ PFU/mL at peak) over 6.1 days.

**Conclusions/Significance:**

Our results suggest that spotted doves, one of the most abundant bird species in China, could be a competent amplifying host for SFTSV and play an important role in its ecology. Between the two SFTSV strains, the strain of the Japanese lineage caused mortality, higher viremia titers in infected birds over a longer time period than did the Chinese strain. Our observations shed light on the ecology of SFTSV, which could benefit the implementation of surveillance and control programs.

## Author summary

Severe fever with thrombocytopenia syndrome virus (SFTSV) is an emerging human pathogen naturally transmitted by ticks. Our recent study has showed that some species of migratory birds, such as swan geese and spotted doves, can be parasitized by *Haemaphysalis longicornis*, an SFTSV vector, and antibodies against the SFTSV detected in these species. These data demonstrate that migratory birds are infected with SFTSV and may also play a role in the infection of ticks with SFTSV. Other studies have reported that migratory bird routes and the distribution of *H*. *longicornis* in East Asia overlap with the geographic distribution of SFTSV. Migratory birds are known to be carriers and transmitters of infectious agents, like the causative agents of influenza, West Nile encephalitis, and Lyme disease. Wild birds often travel long distances carrying various parasites, including ticks, which may be infected with viruses and bacteria. It is therefore reasonable to hypothesize that migratory birds may have played an important role in spreading SFTSV in two potential transmission scenarios: 1) birds are infected with the virus and transmit it back to ticks endemically or in a distal region, or 2) they are carriers of parasitic ticks that are infected with the virus. Here we report an experimental infection of spotted doves (*Streptopelia chinensis*) with two strains of SFTSV, JS2010-14 from a Chinese lineage and JS2014-16 from a Japanese lineage, which represent the main viral genotypes currently circulating in East Asia. We determined that spotted doves are susceptible to SFTSV and that the severity of the viremia was dose and strain dependent. Infection with the strain of JS2014-16 led to a death rate of 12.5% and higher viremia titers in experimentally inoculated birds while doves inoculated with the JS2010-14 strain survived infection with relatively lower virus titers in the blood. These findings provide novel insights for understanding the rapid spread of the virus in a short time span, in particular the SFTSV strains from the Japanese lineage (genotype E).

## Introduction

Severe fever with thrombocytopenia syndrome virus (SFTSV) is a phlebovirus in the family *Phenuiviridae* and causes severe fever with thrombocytopenia syndrome (SFTS), a severe hemorrhagic fever disease in East Asia [1,2]. The disease is characterized by high fever and a drastic reduction of platelets and leukocytes leading multi-organ failure with mortality up to 10% in patients. SFTSV was first isolated from a patient in eastern China in 2010. By the end of 2017, more than 12,000 cases were reported in 23 provinces of China making the disease an important public health concern [3,4,5,6].

The SFTSV is a tick-borne zoonotic virus that has been detected in or isolated from several species of ticks, especially *Haemaphysalis longicornis*, a widely-distributed tick species in East Asia [7,8,9]. SFTSV has a broad spectrum of animal hosts but none of the animals thus far have been confirmed as reservoir hosts. Previous studies conducted in East Asia including China, South Korea, and Japan showed that many domesticated and wild animals were susceptible to SFTSV infection but had no or inconspicuous clinical signs [10,11,12,13]. Additionally, in our previous study we demonstrated that some species of migratory birds, such as swan geese (*Anser cygnoides*) and spotted doves, could be both parasitized by *H*. *longicornis* and infected by SFTSV. These two characteristics demonstrate the potential for these species to contribute to the long-distance spread of SFTSV via migratory flyways [7]. This theory could explain why SFTSV has spread rapidly in China and genetically related viral strains were identified in China, Japan and Korea within a relatively short time span. Experimental infection with SFTSV causes mild clinical disease with moderate viremia levels in some vertebrate animals, which might serve as amplifying hosts in the natural transmission cycle of SFTSV [14,15,16]. However, susceptible avian species and their responses to SFTSV infection has not been established.

Spotted doves are common birds in China. This species is found in most parts of China in summer months, but in winter, most migrate to warmer areas of southern China [17]. Spotted doves are also common birds in Japan and Korea where SFTSV also circulates [18,19]. In this study, we challenged naive spotted doves with two genotypes of SFTSV to establish an avian model of infection. Our objective was to determine the susceptibility of spotted doves to SFTSV infection, examine virulence and duration of viremia to assess the potential role of doves in SFTSV ecology.

## Materials and methods

### Ethics statement

All bird transport, handling, daily husbandry, and study protocols were conducted in strict accordance with the Animal Ethics Procedures and Guidelines of the People’s Republic of China (Regulations for Administration of Affairs Concerning Experimental Animals, China, 1988). Protocols were pre-reviewed and approved by the Ethics Committee of the Jiangsu Provincial Center for Disease Control and Prevention (Certificate No. JSCDCLL [2016]032). Moribund birds and all birds remaining at the end of the study were anesthetized with isoflurane gas and then euthanized with cervical dislocation.

### Sources of viruses and birds

Two SFTSV strains, JS2010-14 of Chinese lineage (hereafter JS2010) and JS2014-16 (hereafter JS2014) of Japanese lineage, were used in the study. Both viral strains were isolated from SFTS cases in Jiangsu province of China in 2010 and 2014, respectively. The spotted doves were purchased from a commercial breeder of the species in China and held for two weeks to acclimate prior to SFTSV challenge. The birds used for the study were determined to be clinically healthy by a qualified veterinarian. Upon arrival, each bird was given a numbered leg band and caged in a biosafety level 3 animal facility. The spotted doves were provided 12 hr light/12 hr darkness, housed in groups of four or eight in wire cages measured at approximately 80 cm (long) x 60 cm (wide) x 60 cm (height) and were provided a commercial seed mix and water *ad libitum*. All birds enrolled in the study were males, 2 months of age and approximately 400 grams in weight.

### Challenge of spotted doves with SFTSV

Two independent challenge studies were conducted. To investigate the susceptibility of spotted doves to SFTSV infection, doves in the first study were randomly assigned to one of four treatment groups: procedural controls (n = 4), and three SFTSV challenge groups, each given a different SFTSV dose: 10^3^ (n = 8), 10^5^ (n = 8), and 10^7^ (n = 8) PFU. This study was replicated for both SFTSV strains, JS2010 and JS2014. The birds in the control groups from each replicate were housed together and separately from the virus challenged groups. On day 0 of the study, control birds were sham inoculated, specifically they were injected subcutaneously (s.c.) with 100 μL of serum-free Dulbecco’s Modified Eagle Medium (DMEM) as previously described [20]. The individual birds in the three SFTSV challenge groups were each inoculated s.c. with 10^7^, 10^5^, or 10^3^ PFU of a low passage (<3) human origin isolate of SFTSV suspended in 100 μL of DMEM according to their group assignment. A bird was considered infected with SFTSV if live virus was isolated from a serum sample at any sampling time point or if the bird developed anti-SFTSV antibodies. Each dove in the virus-inoculated groups was sampled on day 1 through 14 post-inoculation (pi). On each sampling day, 100 μL of blood was collected. Whole blood was allowed to clot for 30 min at room temperature in blood collection tubes and held at 4°C until centrifugation at 2000 x g for 10 min. Serum was collected and diluted in DMEM for a final serum: media dilution of 1:5. The resulting diluted serum samples were stored at -80°C until testing.

Following SFTSV challenge, birds were observed for clinical signs daily over 14 days. Birds that were moribund, as characterized by difficulty perching or other neurological signs, were humanely euthanized. At 14 dpi all birds were anesthetized with isoflurane gas and then euthanized with cervical dislocation.

In the second study, for each SFTSV strain, 12 spotted doves were inoculated with a dose of 10^5^ PFU with an additional four birds inoculated with serum-free DMEM to serve as negative controls. Three birds were selected randomly from the inoculated ones at 2, 4, 7, and 14 dpi and euthanized after blood collection for necropsy to collect heart, liver, lung, spleen, kidney, and brain tissues. A small portion of each organ was collected, weighed, and homogenized in 1 mL of lysis buffer(Qiagen, Germany)using a mini-bead beater instrument (TissueLyser LT, Qiagen, Germany). Real-time RT-PCR was used to quantify SFTSV in the homogenized organs. The negative control birds were euthanized and necropsied at 14 dpi and their organs processed in the same way. The sera from the challenged birds were also tested for the presence of SFTSV and anti-SFTSV antibodies at 2, 4, 7, and 14 dpi by virus isolation and ELISA.

### Virus isolation and titration

Virus titration was performed as described [20] using a 24-well plate for a mini-plaque assay technique to accommodate small sample volumes. A half milliliter of Vero cells (2 x 10^5^ cells/ml) in DMEM was added to each well. The plates were incubated for 4 days at 37°C in an incubator with 5% CO_2_. Sera were individually centrifuged at low speed for clarification. Supernatants were diluted 1:10 in DMEM containing 10% fetal bovine serum. Individually diluted viral inoculum was added to three wells with confluent Vero cell monolayers in the 24-well plate and incubated for 45 min after which the inoculum was removed. One milliliter of complete agarose overlay was added to each well. The 1:10 serum:DMEM samples were screened for the presence of virus by plaque formation and the positives were further titrated on 12-well plates with ten-fold serial dilution for endpoint titration. Cell cultures were examined for plaque formation at 96, 120, and 144 hrs pi and the number of plaques was recorded. Infectious virus titers were calculated as PFU/ml.

### Virus detection by RT-PCR

RNA was extracted from 140 μL of the 1:10 diluted serum samples using the QIAamp Viral RNA Mini kit (Qiagen). RNA was extracted from brain tissuewith the RNeasy Lipid Tissue Mini extraction kits (Qiagen) and from other organs using the RNeasy Mini extraction kit (Qiagen). Real-time RT-PCR was performed using the QuantiTech RT-PCR kit (Qiagen). The primers were designed as previously described and used in a one-step real-time RT-PCR [21]. The forward (S-for)/reverse (S-rev) primers and MGB probe (S-pro) used in the real-time RT-PCR were targeted to the S segment of the viral genome. Conditions for the reaction were as follows: 50°C for 30 min, 95°C for 15 min, 40 cycles at 95°C for 15 sec, and 60°C for 1 min. Amplification and detection were performed with an Applied Biosystems 7500 Real-time PCR system (Applied Biosystems, Foster City, CA). Data were analyzed using the software supplied by the manufacturer.

### Antibody detection

Prior to challenge, we sampled the blood of all birds. All were to be seronegative for specific antibodies to SFTSV by plaque reduction neutralization assay (PRNT). Serum samples were also collected at 14 dpi, prior to euthanasia or at the time of death in the first study. All serum samples were heat-inactivated at 56°C for 30 min and tested for anti-SFTSV antibodies by PRNT on 12-well plates as described [15]. Samples exhibiting a neutralization of ≧90% were considered positive for antibodies to SFTSV (PRNT_90_). Additional sera were tested for both IgG and IgM SFTSV antibodies with a commercial double antigen sandwich ELISA kit from Xinlianxin Biotech (Wuxi, China). The assay was developed for detecting total antibodies specific to SFTSV in various animal species including birds [13]. Positive sera were 2-fold diluted starting at 1:10 for the assay to obtain endpoint titers determined by the cutoff values set by the positive and negative ELISA controls.

### Statistical analysis

All statistical analyses were performed with SPSS 19.0 (SPSS, Chicago, IL) and statistical significance level was set at 0.05. For categorical data, the proportion and 95% confidence interval (CI) were calculated and differences in proportions were compared with the Fisher's exact test. Unless indicated, all tests of proportions or means were two-sided.

## Results

### Susceptibility of spotted doves to SFTSV

**Fig 1:**
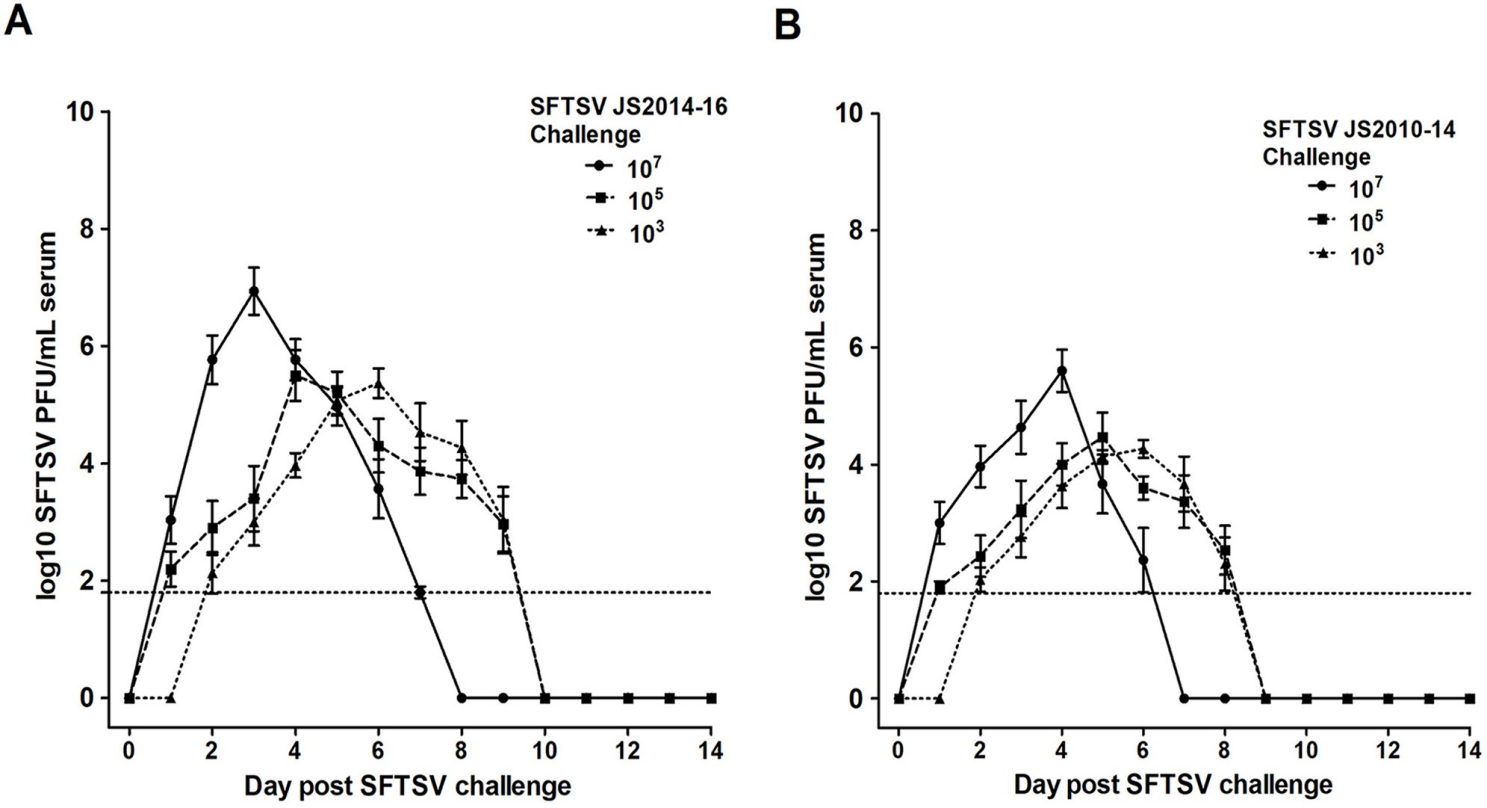
Experimental infection of spotted doves with two SFTSV strains. Error bars represent standard error of the mean log_10_ PFU/mL of serum. The horizontal dashed line indicates a limit of detection of 10^1.8^ PFU/mL. A: Spotted Doves inoculated with 10^7^ plaque forming units (PFU) of SFTSV strain JS2014 (solid line) had higher mean viral loads that peaked earlier than did birds inoculated with 10^5^ (dashed line) or 10^3^ PFU (fine dashed line) of virus. B: Spotted doves inoculated with 10^7^ plaque forming units (PFU) of SFTSV strain JS2010 (solid line) had higher mean viral loads that peaked earlier than did those birds inoculated with 10^5^ (dashed line) or 10^3^ PFU (fine dashed line) of virus.

In the first study, our results demonstrate that spotted doves were infected and developed viremia after inoculation with either viral strain (Table 1, Fig 1). Viremia appeared in all birds challenged with 10^7^ and 10^5^ PFU of either viral strain. When challenged with the dose of 10^3^ PFU, viremia was detected in fewer birds than in the groups challenged at higher doses. When challenged at 10^3^ PFU, more birds were viremic after challenge with strain JS2014 of the Japanese lineage (5/8) than with strain JS2010, of the Chinese lineage (2/8) (Table 1).

**Table 1:** Susceptibility of spotted doves to SFTSV infection is dependent on challenge dose.

Challenge dose (PFU)	JS2014					JS2010				
	No.	Infection** (%)		Mortality (%)	Mean Day* (95% CI)	No.	Infection** (%)		Mortality(%)	Mean Day* (95% CI)
		Viremia	Ab				Viremia	Ab		
10^7^	8	8 (100%)	8 (100%)	1 (12.5%)	2.6 (2.2–3.3)	8	8 (100%)	8 (100%)	0 (0%)	3.7 (2.9–4.4)
10^5^	8	8 (100%)	8 (100%)	0 (0%)	3.7 (2.8–4.2)	8	8 (100%)	8 (100%)	0 (0%)	4.8 (3.7–5.5)
10^3^	8	5 (62.5%)	6 (75%)	0 (0%)	4.6 (3.8–5.3)	8	2 (25%)	4 (50%)	0 (0%)	5.6 (4.9–6.6)

* mean day on which peak viremia occurred (95% CI)

**Infection was determined by viral culture or antibody detection.

We were able to detect SFTSV-specific antibodies in doves challenged with each of the SFTSV strains. In the JS2010 and JS2014 10^3^ PFU challenged groups, two and one additional birds developed anti-SFTSV antibodies without being detected as viremic, respectively. All control birds (n = 4) were negative as determined by viral isolation, viral specific antibody detection, and real-time RT-PCR and none died.

Mortality was observed only in the group of the birds challenged with 10^7^ PFU of the strain JS2014 (1/8, 12.5%) on day 7 pi. No birds died in the groups inoculated with 10^5^ and 10^3^ PFU of either virus strain and in the group given the JS2010 strain at 10^7^ PFU. Our data suggest that the infection of SFTSV in spotted doves was primarily self-limiting and infected birds recovered after a defined period of viremia.

### Dynamics of viremia in spotted doves challenged with SFTSV

Mean SFTSV viremia levels were highest at 3 dpi in the JS2014 10^7^ PFU challenge group (mean = 10^6.9^ PFU/mL, SD 10^0.3^), followed by the 10^5^ PFU group at 4 dpi (mean = 10^5.5^ PFU/mL, SD 10^0.2^), and by the 10^3^ PFU group on day 5 (mean = 10^5.3^ PFU/mL, SD 10^0.2^). The mean SFTSV viremia levels for the JS2010 10^7^, 10^5^, and 10^3^ PFU challenge groups peaked at 4 dpi (mean = 10^5.6^ PFU/mL, SD 10^0.3^), 5 dpi (mean = 10^4.5^ PFU/mL, SD 10^0.4^), and 6 dpi (mean = 10^4.3^ PFU/mL, SD 10^0.2^), respectively. The mean peak day of viremia for individual birds of the JS2014 10^7^ PFU challenge group (mean 2.6 d) occurred significantly earlier compared to the other challenge groups (Table 1) (overall F = 9.2, p<0.01, Tukey's multiple comparisons of 10^7^ mean to 10^5^ and 10^3^ PFU, q = 5.2 and q = 5.1, respectively). With the strain JS2010, the mean peak day of viremia for individual birds in the 10^7^ PFU challenge group was 3.7 d, significantly earlier compared to the other challenge groups (Table 1) (overall F = 10.2, p<0.01, Tukey's multiple comparisons of 10^7^ mean to 10^5^ and 10^3^ PFU, q = 6.2 and q = 5.5, respectively). Viremia detected in birds from both 10^7^ PFU challenge groups fell to the threshold of detection (5–6 days) more rapidly than either the 10^5^ or 10^3^ PFU challenge groups (7–8 days).

### Multi-organ tropism of SFTSV in spotted doves

In the second study, the virus was successfully isolated from sera in 9 of the 12 spotted doves challenged with JS2014 at 10^5^ PFU. SFTSV specific antibodies were detected in all three inoculated birds at 14 dpi while they were negative by viral isolation. There was no mortality. In the JS2010 10^5^ PFU challenge group, 6 of the 12 spotted doves were positive by viral isolation and three birds, negative for viral isolation at 14 dpi, developed anti-SFTSV antibodies.

**Fig 2:**
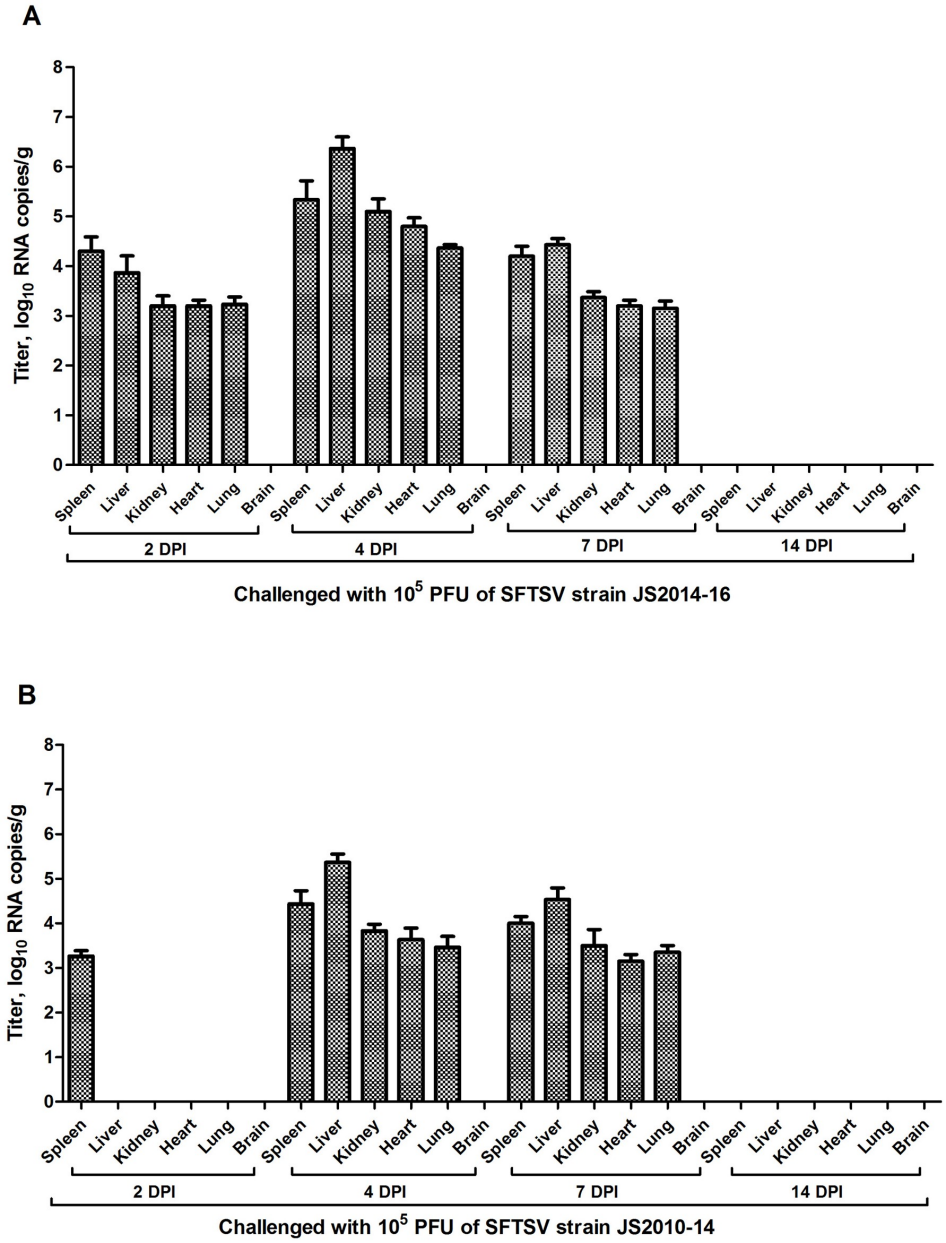
Viral distribution, as determined by RNA copy numbers, in organs from birds experimentally infected with 10^5^ PFU of two SFTSV strains. Viral titers are represented as geometric mean±SD. A detection limit of 0.95 log_10_RNA copies g^-1^ was determined. Spotted doves inoculated with 10^5^ PFU of SFTSV strain JS2014 (A) had higher and earlier mean viral loads than birds inoculated with 10^5^ PFU of SFTSV strain JS2010 (B).

As for temporal viral distribution in organs of the birds challenged with 10^5^ PFU of JS2014, SFTSV was detected by viral isolation or RT-PCR in multiple organs, including kidney, liver, heart, lung, and spleen obtained from each of the three sacrificed birds at 2, 4, and 7 dpi (Fig 2). At 14 dpi, however, SFTSV was no longer detectable by either viral isolation or RT-PCR in any tissues from the three sacrificed birds. For the birds inoculated with 10^5^ PFU of JS2010, SFTSV was detected by RT-PCR only, but not by viral isolation, in the spleen of the three sacrificed birds at 2 dpi. At 4 and 7 dpi, kidney, liver, heart, lung, and spleen were all positive with either viral isolation or RT-PCR in all three sacrificed birds. At 14 dpi, SFTSV was not detected by either method in any tissues (Fig 2). All control birds were negative in either viral isolation or RNA detection.

### Development of SFTSV antibodies in spotted doves

**Fig 3:**
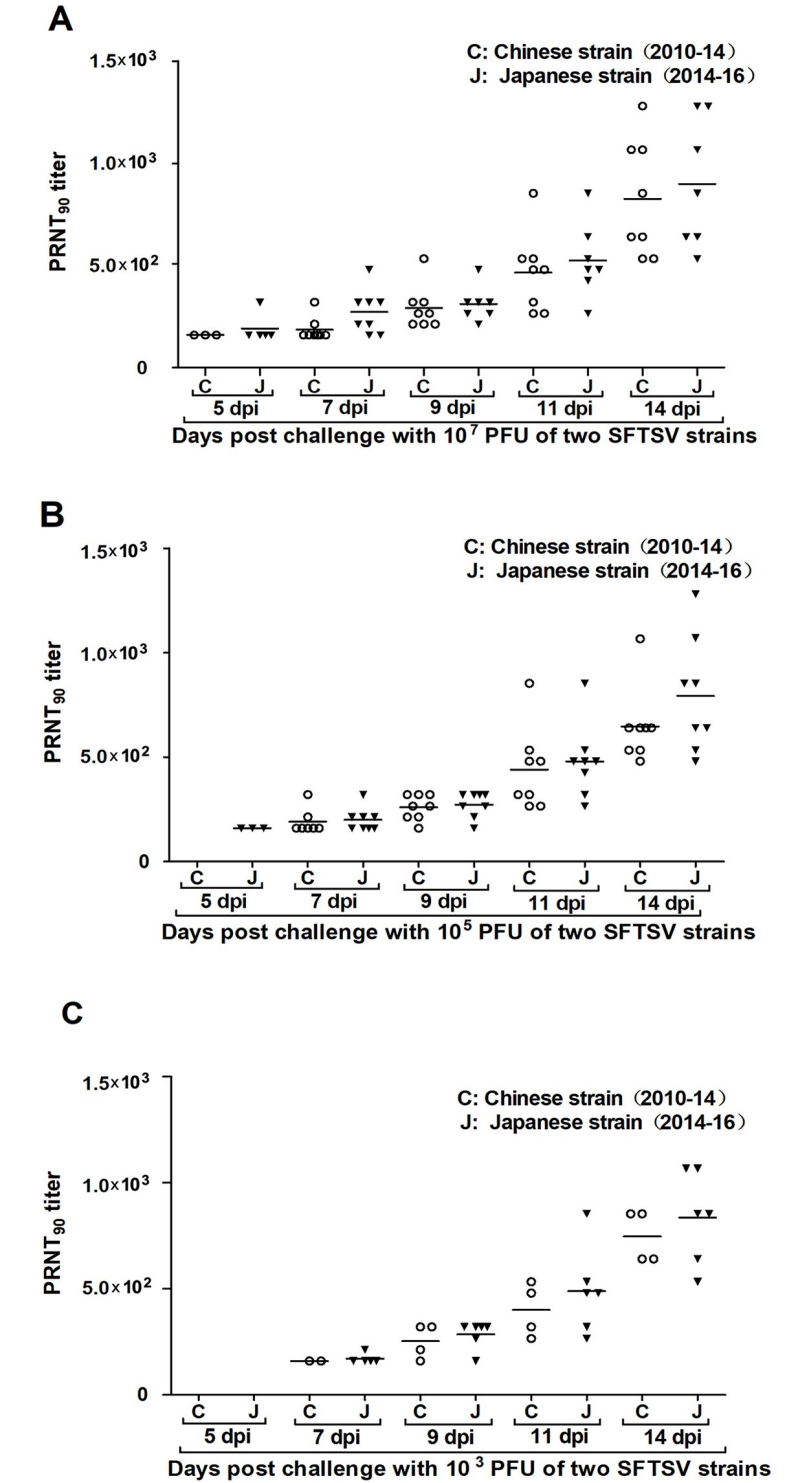
Neutralizing antibody titers in spotted doves infected with different challenge doses of two SFTSV strains. Spotted doves challenged with 10^7^ and 10^5^ PFU of SFTSV (A and B) showed earlier neutralizing antibody than 10^3^ challenge groups (C).

Prior to the study we sampled the blood of all birds that were confirmed to be seronegative for specific antibodies to SFTSV by the PRNT assay. Final serum samples were collected at 14 dpi or at the time of death in the first study, and the antibodies to SFTSV were detected by the PRNT_90_ in 8/8 (100%), 8/8 (100%), and 6/8 (75%) of the infected birds in the groups challenged with 10^7^, 10^5^, and 10^3^ PFU of the strain JS2014, respectively. In the groups challenged with 10^7^, 10^5^, and 10^3^ PFU of the strain JS2010, specific antibodies to SFTSV were detected in 8/8 (100%), 8/8 (100%), and 4/8 (50%) of the infected birds, respectively (Table 1). Neutralizing antibody titers for SFTSV by PRNT_90_ were up to 690–860 in mean (presented as reciprocal of serum dilution) at 14 dpi (Fig 3). Neutralizing antibodies for SFTSV were detected earlier in the spotted doves challenged with 10^7^ and 10^5^ PFU than in birds given 10^3^ PFU (Fig 3).

### Pathogenicity of SFTSV in spotted doves

In the first study, all infected birds underwent a period of anorexia that coincided with detectable viremia in the groups challenged with 10^7^ and 10^5^ PFU of both SFTSV strains. Moreover, one dove challenged with 10^7^ PFU of JS2014 became lethargic, had ruffled feathers and thus was euthanized on day 7. This bird had the highest viremia in the group, (up to 10^7.3^ PFU). In the groups challenged with 10^3^ PFU of either viral strain, only the birds with detectable viremia were anorexic. No other clinical signs were observed.

**Fig 4:**
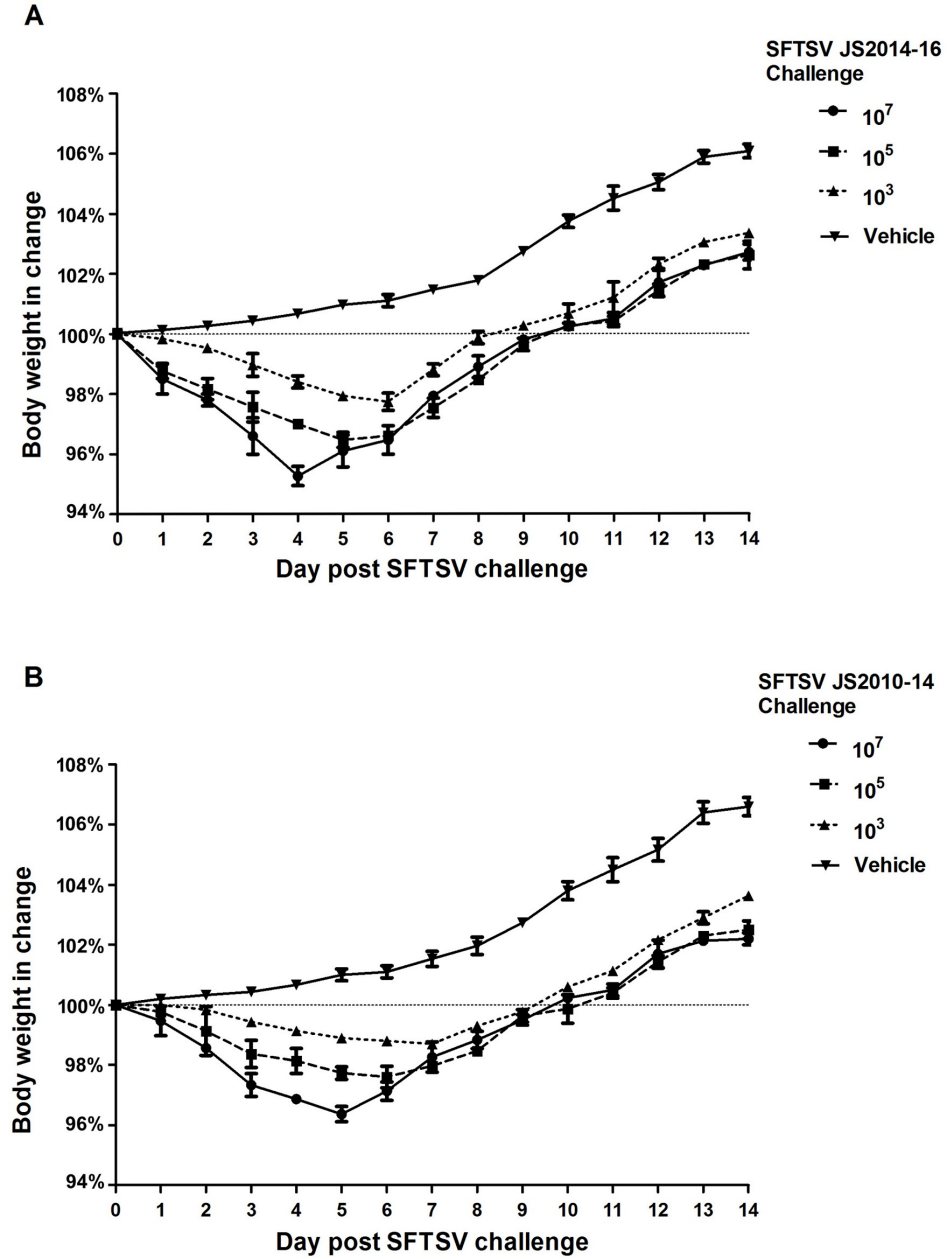
Change in body mass of the spotted doves following SFTSV challenge. Mean changes in body mass and standard error bars are plotted at daily intervals following challenge of the spotted doves. Changes in body mass following SFTSV challenge showed a dose and strain dependent response. Birds challenged with 10^7^ plaque-forming units (PFU) of SFTSV strain JS2014 had earlier and greater losses of body mass than did birds challenged with 10^5^ or 10^3^ PFU of the same SFTSV strain. Birds challenged with SFTSV strain JS2014 had greater losses of body mass than the birds challenged with same dose level of SFTSV strain JS2010.

A dose-related loss of body weight was detected in birds following challenges with either of the SFTSV strains in the first study (Fig 4). The birds challenged with 10^7^ PFU JS2014 had the largest drop in body mass, with an average of 4.7% by 4 dpi. Challenged with 10^5^ and 10^3^ PFU of the strain JS2014, the birds lost 3.5% and 2.3% of average body mass at 5 and 6 dpi, respectively. The birds challenged with 10^7^ PFU of the strain JS2010 showed the greatest body mass loss of 3.6% average at 5 dpi. The doves lost 2.4% and 1.3% of average body mass at 6 and 7 dpi, respectively, when challenged with 10^5^ and 10^3^ PFU of the strain JS2010. The JS2014 strain appeared to cause the earlier and more severe loss of body weight than did the JS2010 strain. Around 10 dpi the mean body weights had either returned to or exceeded their starting levels in all challenge groups.

## Discussion

SFTS is an emerging zoonotic disease which is traced back to reports of an unknown infectious disease in rural areas of Hubei and Henan provinces in central China in 2009 [1]. The causative agent was not initially identified due to similar clinical manifestations caused by *Anaplasma phagocytophilum*, Hantaan virus, and *Rickettsia tsutsugamushi* infections [1,2]. A phlebovirus was finally isolated from a farmer in Henan, China and confirmed as the cause of SFTS [1]. Surveillance data showed that SFTSV spread to 23 provinces in China from 2010 to 2017 [3,4,5,6,22]. Furthermore, SFTS cases have been reported in other Asian countries including South Korea and Japan [23,24].

Several studies on the geographic distribution, genetic diversity, and prevalence of SFTSV genotypes have led to the proposal that there are two major SFTSV lineages, the Chinese and Japanese lineages [25]. Phylogenetically the SFTSV strains are grouped in 5 clades (A, B, C, D and E) based on the sequences of their genome segments (S1 Fig). Clades A, B, C and D are classified as the Chinese lineage, while clade E is classified as the Japanese lineage. SFTSV strains isolated from China include all 5 clades, the strains from South Korea fall into 3 clades (A, D, and E), and all strains from Japan are from only clade E [4,25,26]. At present, the SFTSV strains of clade E are the most widely disseminated in East Asia.

Recent analyses indicate that SFTSV might have originated in the Dabie Mountain area in central China. According to the theory, several decades ago the virus was introduced to Shandong Province from Henan Province, and then to Liaoning Province in Northeastern China, the Zhoushan Archipelago of China, Jeju Island of South Korea, and to Japan from Jiangsu province [26]. Transmission across land and sea must have happened to explain the distribution of SFTSV and particularly the viruses of clade E [26].

Previous studies have found that some domestic animals and wildlife may be infected with SFTSV and might serve as amplifying or reservoir hosts in the natural transmission cycle of SFTSV [11,12,13,14]. Our recent study showed that some species of migratory birds, such as swan geese and spotted doves, can be parasitized by *H*. *longicornis* and infected by SFTSV in nature [7]. Other studies have reported that migratory bird routes and the distribution of *H*. *longicornis* in East Asia overlap with the geographic distribution of SFTSV [27,28]. Migratory birds are known to be carriers and transmitters of infectious agents, like the causative agents of influenza, West Nile encephalitis, Crimean-Congo hemorrhagic fever, and Lyme disease [29,30,31]. Wild birds often travel long distances carrying parasites, including ticks, which may be infected with viruses and bacteria. While birds play a key role in spreading the above-mentioned virus or bacterium, mosquitoes and ticks are involved in the transmission of the agents for West Nile Virus, Crimean-Congo hemorrhagic fever virus, and Lyme spirochetes, respectively. It is reasonable to hypothesize that migratory birds may have an important role in spreading SFTSV in two scenarios, i.e., either the birds are infected directly with the virus or the birds are carriers of parasitic ticks that bear the virus.

Spotted doves are a common migratory species in China. In this study, we challenged spotted doves with Chinese (clade A) and Japanese lineage (clade E) SFTSV strains to establish a bird infection model of SFTSV. We were also interested in examining host susceptibility to infection, viral pathogenicity and duration of viremia, in order to assess the potential roles of spotted doves as a reservoir and/or amplifying hosts of SFTSV. The results showed that the spotted doves were susceptible to both clades of SFTSV. Viremia appeared in all birds challenged with the doses of 10^7^ and 10^5^ PFU of both viral strains. Most of the spotted doves challenged with the dose of 10^3^ PFU were infected and had detectable viremia or SFTSV-specific antibodies. Mortality was observed (1/8, 12.5%) only in the group of the birds challenged at 10^7^ PFU with the clade E virus, or the Japanese strain JS2014, on day 7 pi. No birds died in either the JS2014 10^5^ or 10^3^ PFU challenged groups and in all challenge levels of the Chinese strain JS2010. This suggests that the infection of spotted doves with SFTSV was primarily self-limiting and infected birds mostly recovered after a period of viremia.

Our data in this report indicate that differential susceptibilities to the two clades of viruses may occur in spotted doves. Of the two viral strains tested, JS2014 led to one death and higher viremia titers among infected birds, while JS2010 caused no fatalities and had relatively lower virus titers in the blood of inoculated birds (Fig 1). Although necropsy was not performed on the bird that died, it did have the highest viremia levels of the group prior to death. Therefore, the mortality event was most likely caused by viral infection. We speculate that with higher viremia titers, the birds could transmit the virus to feeding ticks more efficiently. Thus, as a potential amplifying host, the spotted doves may be more efficient in transmitting the Japanese lineage SFTSV. This is consistent with studies on the geographic distribution of SFTSV genotypes, i.e., the SFTSV strains of the Japanese lineage are more widely disseminated geographically, possibly due to its higher replication efficacy in migratory birds such as doves.

To date, only a few mammals have been used as models for the study of SFTSV infection, including mice, goats, hamsters and macaques [10,14,15,16]. To our knowledge, this is the first study to examine spotted doves as a host of SFTSV. The results showed that the spotted doves are susceptible to SFTSV infection, particularly by clade E or the Japanese origin strains, and could be a competent amplifying host species. Thus, these birds may play an important role in the long-distance transmission of the virus. Heartland virus is a phlebovirus closely related to SFTSV. Infection of Heartland virus in field collected avian hosts was not observed [32,33]. However, infection of SFTSV was observed in chickens and some species of field collected birds as recently reported [14,15,16]. Experimental inoculation of chickens with Heartland virus resulted in no viremia and scanty immune responsiveness [32,33]. In contrast, this study demonstrates that the spotted doves can be experimentally infected by SFTSV resulting in detectable viremia and immune responsiveness. A differential susceptibility to closely related phleboviruses appears to occur in birds. The underlying mechanism for the difference, however, remains unknown and deserves further studies.

## Supporting information

S1 Fig Phylogenetic analysis of the SFTSV strains JS2010 and JS2014, compared with other SFTSV strains from different endemic areas. The phylogenetic trees were constructed using the Maximum Likelihood Method with the MEGA5.1 software based on the L segments of SFTSV strains from endemic areas. The reliability values indicated at the branch nodes were determined using 1,000 bootstrap replications. Bootstrap value ≥70 were labeled at nodes. Colored taxon names of the phylogenetic trees represented the SFTSV strains used in this study.
(TIF)
