# Supplementary figures and images for: Susceptibility of spotted doves (Streptopelia chinensis) to experimental infection with the severe fever with thrombocytopenia syndrome phlebovirus
Source: PLoS Negl Trop Dis. 2019 Jul 5;13(7):e0006982. doi: 10.1371/journal.pntd.0006982 (PMC6636776; doi:10.1371/journal.pntd.0006982)

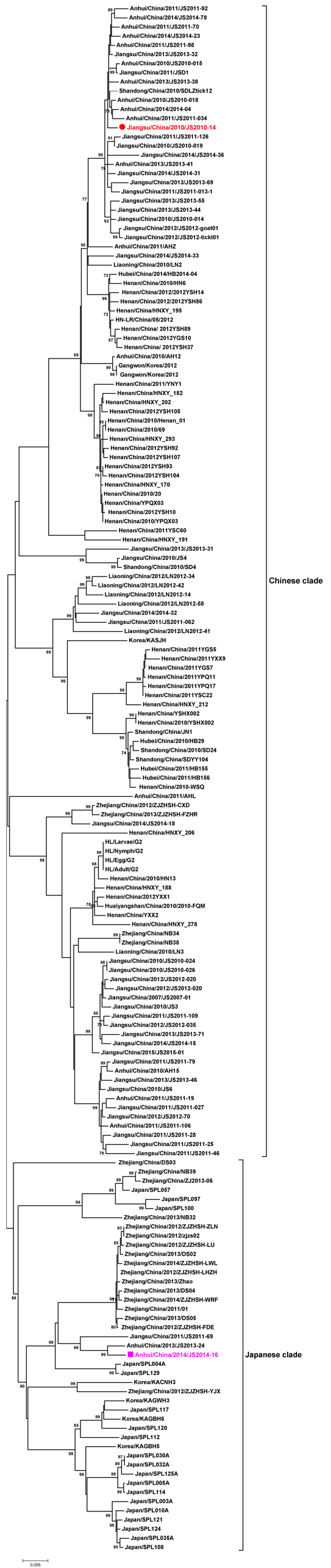

Supplement: S1 Fig — The phylogenetic trees were constructed using the Maximum Likelihood Method with the MEGA5.1 software based on the L segments of SFTSV strains from endemic areas. The reliability values indicated at the branch nodes were determined using 1,000 bootstrap replications. Bootstrap value ≥70 were labeled at nodes. Colored taxon names of the phylogenetic trees represented the SFTSV strains used in this study. (TIF) [file pntd.0006982.s001.tif]
